# The non-pathogenic protozoon *Leishmania tarentolae* interferes with the activation of NLRP3 inflammasome in human cells: new perspectives in the control of inflammation

**DOI:** 10.3389/fimmu.2024.1298275

**Published:** 2024-04-19

**Authors:** Francesca La Rosa, Ilaria Varotto-Boccazzi, Marina Saresella, Ivana Marventano, Giulia Maria Cattaneo, Ambra Hernis, Federica Piancone, Domenico Otranto, Sara Epis, Claudio Bandi, Mario Clerici

**Affiliations:** ^1^ IRCCS Fondazione Don Carlo Gnocchi, Milan, Italy; ^2^ Department of Biosciences, University of Milan, Milan, Italy; ^3^ Pediatric Clinical Research Center ‘Romeo ed Enrica Invernizzi’, University of Milan, Milan, Italy; ^4^ Department of Veterinary Medicine, University of Bari, Valenzano, Italy; ^5^ Faculty of Veterinary Sciences, Bu-Ali Sina University, Hamedan, Iran; ^6^ Department of Pathophysiology and Transplantation, University of Milan, Milan, Italy

**Keywords:** immunity responses, inflammasome pathway, *Leishmania tarentolae*, NLRP3, inflammation

## Abstract

**Background:**

Innate immune responses against infectious agents can act as triggers of inflammatory diseases. On the other hand, various pathogens have developed mechanisms for the evasion of the immune response, based on an inhibition of innate immunity and inflammatory responses. Inflammatory diseases could thus be controlled through the administration of pathogens or pathogen-derived molecules, capable of interfering with the mechanisms at the basis of inflammation. In this framework, the NLRP3 inflammasome is an important component in innate antimicrobial responses and a major player in the inflammatory disease. Parasites of the genus *Leishmania* are master manipulators of innate immune mechanisms, and different species have been shown to inhibit inflammasome formation. However, the exploitation of pathogenic *Leishmania* species as blockers of NLRP3-based inflammatory diseases poses safety concerns.

**Methods:**

To circumvent safety issues associated with pathogenic parasites, we focused on *Leishmania tarentolae*, a species of *Leishmania* that is not infectious to humans. Because NLRP3 typically develops in macrophages, in response to the detection and engulfment microorganisms, we performed our experiments on a monocyte-macrophage cell line (THP-1), either wild type or knockout for ASC, a key component of NLRP3 formation, with determination of cytokines and other markers of inflammation.

**Results:**

*L. tarentolae* was shown to possess the capability of dampening the formation of NLRP3 inflammasome and the consequent expression of pro-inflammatory molecules, with minor differences compared to effects of pathogenic *Leishmania* species.

**Conclusion:**

The non-pathogenic *L. tarentolae* appears a promising pro-biotic microbe with anti-inflammatory properties or a source of immune modulating cellular fractions or molecules, capable of interfering with the formation of the NLRP3 inflammasome.

## 1 Introduction

Human leishmaniases are a group of vector-borne infectious diseases caused by protozoan parasites of the genus *Leishmania*, which are transmitted by sand flies hematophagous insects of the subfamily Phlebotominae (1). These diseases are widespread in the tropical, sub-tropical, and temperate zones of the world, causing every year an estimated one million cases of cutaneous leishmaniasis and 90,000 cases of the visceral form of the disease (2). Over 20 species of *Leishmania* cause leishmaniases in mammalian hosts. In humans, most cases are caused by *Leishmania donovani*, *L. major*, *L. tropica*, *L. guyanensis*, *L. braziliensis*, *L. mexicana*, and *L. infantum*, with the latter being responsible also for leishmaniasis in dogs. However, not all *Leishmania* parasites infect mammals. Among others, *Leishmania tarentolae* (Subgenus *Sauroleishmania*) infects lizards (e.g., *Tarentola mauritanica* and *Podarcis siculus*) and has been studied in-depth, mainly for its potential applications in the biotechnological and biomedical fields (3, 4). The lack of pathogenicity of *L. tarentolae* toward humans has been associated with the absence of the major virulence factor amastigote-specific protein A2, which is responsible for parasite virulence and visceralization in pathogenic leishmaniae (5, 6). In *in vitro* experiments on mammalian macrophage cells, *L. tarentolae* displayed a limited survival time (up to 24 h), and there is no evidence for any disease caused by this parasite in humans or other mammals (7, 8). Therefore, *L. tarentolae* has been classified as a biosafety level I microorganism (9). Based on the absence of pathogenicity in mammals, *L. tarentolae* has been proposed as a vaccine platform, suitable to be exploited for the production of recombinant antigens and for their delivery to antigen presenting cells (10–13).

Leishmaniases typically cause immune-mediated disorders (e.g., immune-complex–associated pathology), in which the first interactions of the parasite with cells and receptors of innate immunity play a role into the outcome of the infection (14). Nod-like receptor (NLR) family proteins are crucial sensors of microorganisms in innate immunity. Detection of microbial pathogens by NLRs can be direct, through the interaction with pathogen-associated molecular patterns (PAMPs), such as flagellin and peptidoglycan-associated residues, or indirect, through the sensing of signals of cellular damage or stress. Among all NLRs, the NLR pyrin domain–containing 3 protein (NLRP3) is the best characterized. NLRP3 is a key component of a multimolecular structure, known as the NLRP3 inflammasome. Multiple steps are required for the formation of this inflammasome. The activation of the NLRP3 protein induces the assembling of the corresponding inflammasome complex (NLRP3 inflammasome), which also contains the adaptor protein ASC [apoptosis-associated speck-like protein containing C-terminal caspase recruitment domain (CARD)] and an active caspase site. This process requires that procaspase proteins are converted into active forms. This involves the activation of procaspase-1 in the so-called canonical inflammasome formation or the activation of procaspase-11 (in mice) or procaspase-4,5 (in humans) in non-canonical inflammasome formation (15). Two signals are required to determine a complete NLRP3 inflammasome formation and then interleukin-1β (IL-1β) and/or IL-18 secretion. The first one is called priming and induces the expression of the precursors of inflammatory cytokines: pro–IL-1β and pro–IL-18. This process can be triggered by the binding of PAMPs to Toll-Like Receptor 2 (TLR2) and Toll-Like Receptor 4 (TLR4), with induction of transcription nuclear factor–κB (NF-κB). A second signal is then required, to complete the assembly of the NLRP3 inflammasome complex. This involves the sensing of ion flux, ATP, or uric acid, and/or other signs of cellular damage or dysfunction, such as reactive oxygen species. This second signal plays a role in the assembly of the multimolecular complex that comprises ASC and the caspase enzyme, which then determines the cleavage of pro–IL-1β and pro–IL-18 into their bioactive, mature, inflammatory cytokines, and the cleavage of gasdermin D (GSDMD) (16). The N-terminal domain of GSDMD (p30) determines the formation of pores in the plasma membrane, which are associated with an inflammatory form of cell death called pyroptosis (17, 18) that facilitates the release of IL-1β and IL-18.

In response to *Leishmania*, the inflammasome activation involves multiple steps: 1) *Leishmania* is detected by the Dectin-1 receptor and other C-type Lectin receptors; 2) this induces the production of Reactive oxygen species (ROS) via the syk kinase; and 3) ROS finally determine NLRP3 activation (19). A non-canonical activation of NLRP3 by the *Leishmania* lipophosphoglycan (LPG), which triggers mice caspase-11 and human caspase 4/5, has been described as well (20).

To allow the establishment of successful infection, nevertheless, *Leishmania* cells downregulate inflammasome activation to promote their own survival. *L. donovani*–mediated inhibition of the NLRP3 inflammasome was shown to be mediated by two different negative regulators, A20 and UCP2 (mitochondrial uncoupling protein 2). A reduced expression of these proteins results in the control of *Leishmania* infection in mice (21). Another protein with an important role in suppressing NLRP3 inflammasome activation is the metalloprotease GP63, which inhibits the formation of inflammasome through NLRP3 cleavage (22). A study showed that *L. infantum* dampens NLRP3-activation, reducing IL-1ß release and ASC-speck formation, thus favoring an anti-inflammatory milieu (23).

The capability of *Leishmania* spp. to dampen NLRP3 inflammasome formation suggests that these microorganisms might be investigated for their therapeutic potential, in the area of inflammation-based diseases, similarly to parasitic nematodes, that have been proposed as agents to cure immune-mediated disorders (24, 25). However, the use of pathogenic *Leishmania* species as potential therapeutic agents (or as source of therapeutic molecules) poses safety issues at different levels, from the preclinical to the clinical research phases and applications. To avoid these problems, we investigated the immunomodulatory effects of *L. tarentolae*, in *in vitro* experiments aimed at determining whether this non-pathogenic microorganism is capable of dampening NLRP3 activation, similarly to pathogenic species.

## 2 Materials and methods

### 2.1 Cells

Human leukemic cell line (THP-1) wild-type (WT) (IZSLER, Istituto Zooprofilattico Sperimentale della Lombardia e dell’Emilia Romagna, IT) and ASC knockout (KO) THP-1 cells (Invogen, San Diego, USA) were maintained in Roswell Park Memorial Institute medium (RPMI 1640) (EuroClone, Pero, Italy) supplemented with 10% heat-inactivated fetal bovine serum (FBS) (EuroClone), 1% glutamine (EuroClone), 1% penicillin-streptomycin (EuroClone), and Normocin (100 µg/mL) at 37°C in 5% CO_2_. Culture medium was changed three times a week. Cells were seeded in 12-well plates (5 × 10^5^ cells per well); in order to achieve differentiation into macrophages (THP-1–derived Macrophage_dM), phorbol 12-myristate 13-acetate (PMA; 10 ng/mL; Sigma-Aldrich, St. Louis, USA) was added to the wells for 12 h at 37°C in 5% CO_2_.

### 2.2 Parasite culture

Three strains of *L. tarentolae* were tested in this study: i) *L. tarentolae* laboratory strain P10 (Lt-P10) (Jena Bioscience); this commercial strain was derived from the *L. tarentolae* (TAR) strain, which was isolated from the gecko *Tarentola mauritanica* in 1921, and has thus been maintained in laboratory for over 80 years; ii) RTAR/IT/21/RI325, isolated from the gecko *T. mauritanica* (Lt-RI325) (26); and iii) ISER/IT/21/SF178 (Lt-W), isolated from a female sand fly *Sergentomyia minuta* (26). The latter two strains had thus been maintained in the laboratory for less than 2 years. As detailed in the Results section, in a first phase of the study, the experiments were performed using the three strains. Because we did not observe any significant difference between the results obtained with the different strains, we concluded our study only on the commercial Lt-P10 strain.

Lt-P10 promastigotes were cultured in Brain Heart Infusion liquid medium (Sigma-Aldrich, St. Louis, USA) supplemented with porcine hemin (5 μg/mL; Jena Bioscience, Jena, Germany) and 1% penicillin-streptomycin (EuroClone) at 26°C in the dark and under aerated condition. Lt-RI325 and Lt-W promastigotes were maintained in Schneider’s Drosophila medium (ThermoFisher, MA, USA) supplemented with 10% heat-inactivated FBS and 1% penicillin-streptomycin (EuroClone) at 26°C under aerated condition. For culture maintenance, *Leishmania* strains were diluted into fresh medium twice a week.

### 2.3 THP-1 infection

THP-1–derived macrophage cells (THP-1dM) (5 × 10^5^ cells per well) were incubated with stationary phase *L. tarentolae* promastigotes (10 parasites: one cell) for 4 h at 37°C, 5% CO_2_ to allow *Leishmania* internalization (8). Non-internalized promastigotes were removed with two washes with Phosphate-buffered saline (PBS); cells were then incubated for 24 h in medium supplemented with lipopolysaccharide (LPS) (10 ng/mL) (Sigma-Aldrich) plus Nigericin (Nig) (1.34 µM) (Invivogen, USA). At the end of incubation, THP-1dM cells were washed with PBS, harvested by adding 60 μL per well of Accutase (Capricorn Scientific, Ebsdorfergrund, Germany) for 10 min at room temperature (RT) and centrifuged for 10 min at 1,500 rcf. The collected pellet has been used for RNA extraction and for imaging flow analysis (see below). Supernatants of cell culture were collected and frozen at −80°C.

Quantification of lactate dehydrogenase (LDH) released into the medium of cultured cells was effected on WT THP-1dMs, as a proxy of the cellular damages potentially associated with the infection by different *Leishmania* strains. LDH activity was measured in supernatants by LDH assay kit (cod: ab102526) (Abcam, Cambridge, UK), on THP-1 WT and KO cells according to the manufacturer’s recommendations. Measure output was recorded immediately at optical density (OD) of 450 nm on a microplate reader in a kinetic mode choosing two time points (OD at T2-OD at T1) in a linear range. Activity of LDH in the test samples was calculated as: *LDH Acitivity* = (*B/ΔT × V*) ∗ *D* = *nmol min* / / *ml* = *mU mL* /, where B = amount of NADH in sample well calculated from standard curve (nmol), T = reaction time (min) T2-T1, V = original sample volume added into the reaction well (mL), D = sample dilution factor, NADH molecular weight = 763 g/mol, and one-unit LDH = amount of enzyme that catalyzes the conversion of lactate to pyruvate to generate 1.0 μmol of NADH per minute at pH 8.8 at 37°C. LDH internal positive control was included.

In order to evaluate the capability of *L. tarentolae* strains to be phagocytosed by THP-1 macrophages after 4 h, cells were collected, washed with PBS, and finally cytocentrifuged (Cytospin, Hettich, Kirchlengern, Germany) on a slide according to (13). Cells were then fixed with methanol and stained with Giemsa solution (Sigma-Aldrich). The number of infected macrophages per 100 cells counted has been obtained after observation of ten areas of two slides per treatment with an optical microscope (100×).

### 2.4 RNA extraction and reverse transcription

Total RNA was extracted from unstimulated or infected THP-1 cells (5 × 10^5^ cells per well) (see above) using a column-based kit (miRNeasy Mini Kit, Qiagen GmbH, Hilden, Germany) according to the manufacturer’s protocol. RNA concentration was determined by a spectrophotometer (Nanoview plusTM, GE Healthcare, Little Chalfont, UK). Purity was determined as the 260/230 nm absorbance ratio by spectrophotometer (GE NanoVue Plus, LabMakelaar Be JE Zevenhuizen (ZH) Nederland), with the expected values between the range of 1.8 and 2.0 (see **Supplementary Table 1**). RNA was treated with TURBO DNA-free DNAse (Ambion Inc., Austin, TX, US) and was quantified by Qubit (ThermoFisher Scientific). One microgram of RNA was reverse-transcribed into first-strand cDNA using an RT2 First Strand kit (Qiagen, Hilden, Germany) according to the manufacturer’s instructions.

### 2.5 Quantitative real-time RT-PCR

All primers (NLRP3, ASC, caspase-1, caspase-5, caspase-8, IL-1β, and IL-18) (Qiagen GmbH, Hilden, Germany) were cDNA-specific. Samples were evaluated for glyceraldehyde 3-phosphate-dehydrogenase (GAPDH) expression by real-time PCR to test the quality of RNA. Results were expressed as ΔΔCt (where Ct is the cycle threshold) and are presented as ratios between the target gene and the GAPDH housekeeping mRNA. A Bio-Rad CFX Real-Time PCR instrument (Bio-Rad, Hercules, California, USA) with RT2 SYBR Green qPCR mastermix (Qiagen) was used to perform quantitative PCR (qPCR). Results are expressed as the fold changes between stimulated/unstimulated condition. Heat maps were generated and genes hierarchically clustered by Euclidean distance and single linkage using TIGR MultiExperiment Viewer (MeV) v4.9.

### 2.6 Image stream analysis by FlowSight AMNIS

THP-1dM cells incubated with *Leishmania* strains and stimulated with LPS + Nig were collected and labeled for imaging flow analysis. Briefly, cells were permeabilized with 100 μL of Saponine in PBS (0.1%) (Life Science VWR, Lutterworth, UK) and stained with 3 μL (25 μg/mL) of PE-anti human ASC (BioLegend, San Diego, USA) for 1 h at RT in the dark. Cells were then washed with PBS, centrifuged at 1,500 rcf for 10 min, fixed with 60 μL of paraformaldehyde (PFA) in PBS (1%) (BDH, UK), and stored at 4°C until FlowSight AMNIS analyses.

The analysis of ASC speck formation was analyzed by IDEAS analysis software (Cytek Biosciences, Fremont, California) as previously described (27); ASC expression was performed by internalization feature utilizing a mask representing the whole cell, defined by the bright-field (BF) image, and an internal mask defined by eroding the whole cell mask. The same mask of internalization allows the differentiation of diffuse or spot speck fluorescence inside the cells. Specifically, threshold mask was used to separate all ASC-positive cells in “ASC-speck spot cells” or “ASC-diffuse cells” by the different diameter of the spot area: in ASC-speck spot cells, the spot shows a small area and high max pixel, differently from ASC-diffuse cells.

### 2.7 ASC expression by immunofluorescence assays

ASC expression and *Leishmania* internalization were simultaneously determined by immunofluorescence after 24 h of infection. THP-1dM cells incubated with Lt-P10 and stimulated with LPS + Nig were collected, cytocentrifuged on a slide, fixed with PFA in PBS (1%) (BDH, UK), and stained with phycoerythrin (PE) anti-human ASC mAb (25 μg/mL, BioLegend) and 1 μL of mouse anti-*Leishmania* Ab in a solution of Saponine in PBS (0.1%) (Life Science VWR) for 1 h at RT in the dark. After two PBS washes, an Alexa Fluor 488–conjugated anti-mouse Immunoglobulin G (IgG) secondary Ab 1:1000 (ThermoFisher) was added for 1 h at RT. Cells were then mounted with ProLong Gold Antifade Mountant with 4’,6-Diamidino-2-Phenylindole (DAPI; Invitrogen), covered with a coverslip and observed under a fluorescence microscopy Leica DMi8 with Thunder imaging systems with 40× objective.

### 2.8 Cytokine and caspase quantification

Simple Plex Assays for IL-18 (SPCKB-PS-000501), IL-1β (SPCKB-PS-000216), and caspase-1 (p20 subunit) (SPCKB-PS-003613) were run by automated immunoassay system (ELLA) (Biotech, Italy), a microfluidic cartridge that automatizes all steps of the immunoassay. Supernatants collected by stimulated and unstimulated cell culture were centrifuged to remove particulates and analyzed according to the manufacturer’s instruction. Each cartridge is composed of channels that contain glass nanoreactors (GNRs), which are the core of a Simple Plex immunoassay. Each channel of the cartridge contains three GNRs coated with a capture antibody to obtain triplicates. The limit of detection (LOD) was as follows: ASC, 1.95 pg/mL; IL-1β, 0.064 pg/mL; IL-18, 0.2 pg/mL; and caspase-1, 0.04 pg/mL. LOD was calculated by adding three standard deviations (SDs) to the mean background signal determined from multiple runs.

Caspase-5 (cod: MBS094264) and caspase-8 (cod MBS260539) (MyBioSource, Inc.) were measured by sandwich immunoassays (ELISA) according to the manufacturer’s recommendations (MyBioSource, Inc., San Diego CA). A plate reader (Sunrise, Tecan, Mannedorf, CH) was used, and ODs were determined at 450/620 nm. All experiments were performed in triplicates.

### 2.9 Immunofluorescent staining and analysis by flow cytometry

For the analysis of cytokine-secreting or intracellular protein-expressing cells, THP-1dMs were incubated with *Leishmania* for 4 h; then, the cells were incubated for 24 h with stimuli. Then, the cells were washed and fixed (Fix and Perm cell permeabilization kits; Caltag Laboratories, Burlingame, CA, USA) for 15 min at RT in the dark. Cells were then washed and resuspended in permeabilization reagent (Fix and Perm kits) with Tumour Necrosis Factor-alpha (TNF-α), Transforming growth factor-beta (TGF-β) IL-10–, IL-2–, and IL-6–specific mAbs and incubated for 1 h at RT in the dark. Cells were then washed with PBS, centrifuged at 1,500 rcf for 10 min, fixed with 400 μL of PFA in PBS (1%) (BDH, UK), and stored at 4°C until analyses.

The following mAbs were used in this study: PE-labeled anti-human anti–TNF-α–carboxyfluo-rescein (clone 6401, mouse IgG1, R&D Systems), PE-labeled anti-human IL-2 (clone 5334, mouse IgG1, R&D Systems), PE-labeled anti-human IL-6 (clone1936, mouse IgG2B, R&D Systems), PE-labeled anti-human IL-10 (clone JES3-19F1, rat, BD Biosciences), and allophycocyanin-labeled anti-human TGF-b (clone 27232, mouse IgG1, R&D Systems).

Analyses were performed using a Beckman-Coulter GALLIOS flow cytometer equipped with a 22-mW Blue Solid-State Diode laser operating at 488 nm and with a 25-mW Red Solid State Diode laser operating at 638 nm and interfaced with Kaluza analysis software. Flow cytometry compensation was performed using the fluorescence-minus-one (FMO) control approach. Briefly, all antibody conjugates in the experiment are included except the one that is controlled for. The FMO measures the spread of fluorescence from the other staining parameters into the channel of interest, determining the threshold for positive staining.

### 2.10 Statistical analysis

Kolmogorov-Smirnov test was used to verify the normal distribution of the data. Parametric analysis of variance (one-way ANOVA) was initially performed to evaluate *Leishmania* phagocytosis, cytokines, caspase, LDH production, and percentage of ASC-speck–positive cells. Two-tailed paired t-test was performed for gene expression analyses and protein detection by flow cytometry. Results of ANOVA models are shown in the figures as means and SDs. *Post-hoc* comparisons were run using t-tests with Tukey’s honestly significant difference procedure. Data analysis was performed by MEDCALC software (v.14.10.2, Ostend, Belgium) statistical packages. Graphs were obtained using Graph-pad (8.4 version). Results were considered to be statistically significant if surviving the p  <  0.05 threshold.

## 3 Results

### 3.1 *Leishmania* internalization by WT THP-1dMs and KO THP-1dM cells

**Figure 1:**
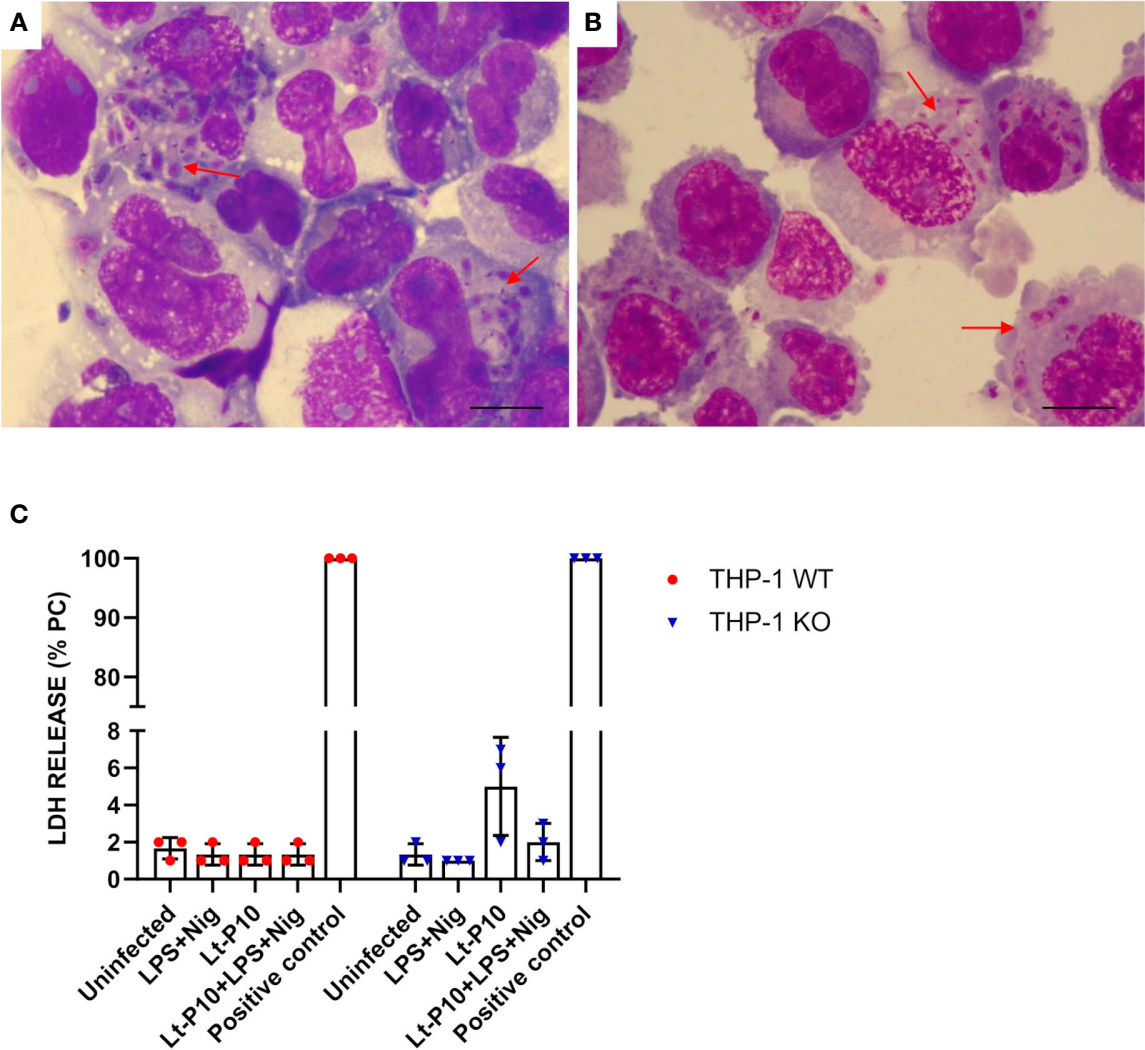
Phagocytosis of *L. tarentolae*–P10 strain by THP-1 cells after 4 h of incubation. THP-1 WT and THP-1 KO macrophages were incubated with Lt-P10 at 1:10 ratio (THP-1:*Leishmania*) for 4 h. Cells were then fixed, stained with Giemsa solution, and observed under an optical microscope (100×). Giemsa smears of Lt-P10 incubated with THP-1 WT **(A)** and with THP-1 KO **(B)** are shown. Red arrows indicate internalized promastigotes inside the cells. Bar, 10 μm. Quantification of lactate dehydrogenase (LDH) released by THP-1 WT or THP-1 ASC-KO cells was carried out on uninfected cells, cells infected with Lt-P10 strain, and uninfected cells stimulated with LPS + Nig **(C)**. Results are expressed as a percentage of internal LDH positive control (PC). Bars show mean ± SD. No significant differences were observed (p > 0.05).

The capability of *L. tarentolae* strains to be internalized by WT THP-1dM cells was evaluated in experiments of co-incubation. WT THP-1dM cells were infected with parasites at a Multiplicity of Infection (MOI) of 10 (10 parasites: one THP-1) for 4 h (these parameters were defined in preliminary experiments and according to published protocols). At the end of the 4 h, cells were collected, and Giemsa smears were prepared in order to calculate the infection rate. All the strains were efficiently phagocytosed, with internalized promastigotes of *Leishmania* being clearly visible inside WT THP-1dM cells (**Figure 1A**, **Supplementary Figures 1A–C**). The infection rate expressed as the percentage of cells with at least one intracellular *Leishmania* was determined for each strain and corresponded to 44%, 36%, and 33%, respectively, for Lt-P10, Lt-RI325, and Lt-W, with no significant differences between strains (**Supplementary Figure 1D**). The phagocytosis of *Leishmania* was also tested on KO THP-1 cells, but, because no differences were observed between the three strains on WT THP-1, we decided to evaluate this parameter using the Lt-P10 strain alone. Results showed that Lt-P10 internalization by KO THP-1dM cells was 41%, a percentage comparable to that obtained with WT THP-1dM cells (**Supplementary Figure 1D**, **Figure 1B**). The choice to focus the experiments only on Lt-P10 strain was also motivated by the fact that this strain, unlike the others (Lt-RI325, Lt-W) isolated in the field, is a commercial strain that has been widely available for years. Additionally, the Lt-P10 strain has already been used for different biotechnological applications, and, therefore, there is a more detailed bibliography, available protocols and sequenced genome, compared to more recent strains isolated in the field.

As a proxy of the cellular damages potentially associated with infection by different *Leishmania* strains, a LDH assay was effected on WT THP-1dM cells co-incubated with Lt-P10, Lt-RI325, and Lt-W. No significant differences in LDH release were observed between *Leishmania* co-incubated cells compared to control, uninfected cells, suggesting that *in vitro Leishmania* infection does not cause cellular damage in our experimental setting (**Supplementary Figure 1E**). The same test was also performed on KO THP-1 cells co-incubated with Lt-P10 strain. A higher percentage of LDH release was observed when the cells were infected with Lt-P10 compared with other stimuli, but the difference is not statistically significant (**Figure 1C**). In these assays, LDH release in control cells, stimulated with LPS + Nig, was rather limited, as compared to results reported in the literature [e.g (28)]. This can be explained considering the differences in the experimental settings: cells used in the experiments; concentration of the stimulating molecules (LPS and Nig); substrate used in LDH determination; etc.

### 3.2 Inflammasome related genes in *L. tarentolae*–infected WT THP-1dMs and KO THP-1dM cells

**Figure 2:**
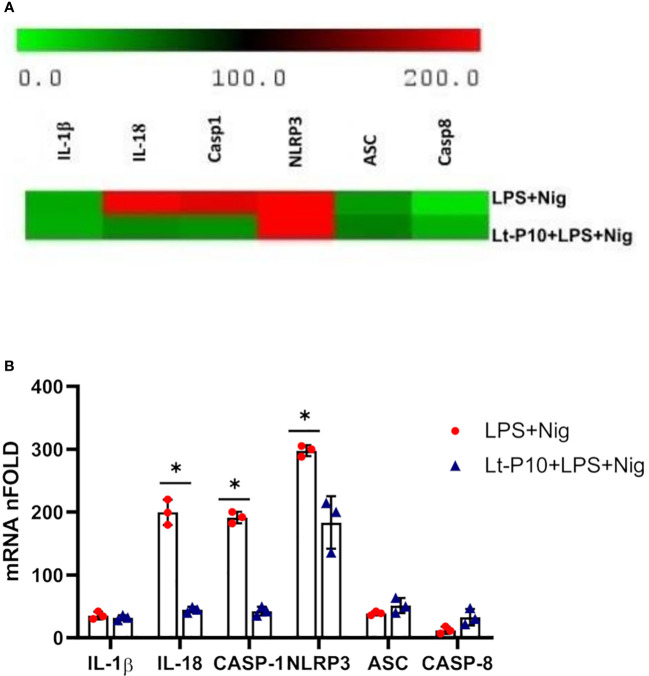
NLRP3 inflammasome gene expression evaluated in THP-1 cells stimulated with LPS + Nig in the presence/absence of *L. tarentolae* P10. THP-1 cells were incubated with Lt-P10 for 4 h at 1:10 ratio and then treated with LPS + Nig for 24 h, and, then, the pellet was collected for RNA extraction. IL-1β, IL-18, caspase-1, NLRP3, ASC, and caspase-8 mRNA expression calculated relative to GAPDH housekeeping gene is shown. Heat maps of log2 fold using changes (software analysis by TIGR MultiExperiment Viewer (MeV) v4.9) are presented **(A)**. Bars show mean ± SD. Results are indicated as fold change expression from the unstimulated samples of three independent experiments **(B)**. Statistical significance differences are indicated. *p < 0.05.

To investigate the expression of genes associated with activation of NLRP3 inflammasome after infection with *L. tarentolae*, a co-incubation experiment has been carried out. Parasites were incubated with macrophages for 4 h and stimulated with LPS + Nig as described above. Then, cell pellets were collected for RNA extraction. NLRP3, ASC, IL-1β, IL-18, caspase-1, and caspase-8 mRNA expression was evaluated as fold change between target genes and the housekeeping gene (GAPDH). Data obtained on WT THP-1dM cells that had been Lt-P10–infected and LPS + Nig–stimulated showed that *L. tarentolae* infection results in a significant reduction of NLRP3, caspase-1, and IL-18 mRNA (p = 0.048, p = 0.03, and p = 0.03, respectively) compared to results obtained in cells that had not been infected (**Figures 2A, B**).

No significant differences between the two culture systems (LPS + Nig stimulation in the presence/absence of infection) were observed for IL-1β and ASC mRNA expression. In contrast with these results, caspase-8 mRNA expression was increased by *L. tarentolae* infection (**Figures 2A, B**). As regards NLRP3, IL-1β, IL-18, caspase-1, and caspase-8 mRNA expression in ASC KO THP-dM cells, no differences were observed between unstimulated and LPS + Nig–stimulated in the presence/absence of infection (data not shown).

### 3.3 ASC-specks formation in response to *L. tarentolae* infection in WT THP-1dM cells

Activation of all inflammasomes requires caspase-1 to be triggered by ASC, generating a large protein complex, which is termed “speck.” ASC specks can be observed as “spots” and reach a size of around 1 μm; in most cells, only one speck forms upon inflammasome activation.

**Figure 3:**
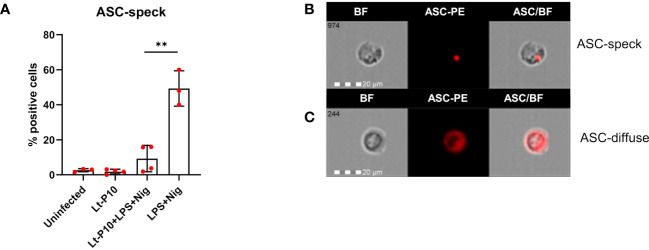
ASC formation in THP-1–derived macrophages. Wild-type cells were incubated with Lt-P10 for 4 h at 1:10 ratio, and, then, the cells were stimulated with LPS + Nig for 24 h. Cells were collected and ASC-PE–stained. The percentage of positive cells for ASC-speck formation in THP-1dM WT cells uninfected or incubated with Lt-P10 and treated with or without LPS + Nig is shown in the graph **(A)**. Data are representative of three independent experiments and expressed as mean ± SD. Statistical significance differences are indicated. **p < 0.01. Identification of ASC-speck cells **(B)** and ASC-diffuse cells **(C)** are shown in the picture. The first column shows cells in bright field (BF), the second column shows ASC-PE fluorescence, and the third column shows fluorescence of ASC merged with BF (IDEA software). The percentage of ASC-speck–positive cells was performed using the same mask of internalization feature differentiating spot (speck) or diffuse fluorescence inside of cells (DF): threshold mask was used to separate all ASC-positive cells population in ASC-speck spot cells or ASC-diffuse cells by the different diameter of the spot area. In ASC-speck cell, the spot shows a small area and high max pixel; conversely, in ASC-diffuse cell, the fluorescence shows a large area and low max pixel.

Results showed that ASC-speck formation was significantly reduced when THP-1 cells, incubated with Lt-P10 and stimulated with LPS + Nig, were compared to LPS + Nig–stimulated but uninfected cell cultures (percentage of cells containing spots: 9.81% vs. 45.62%; p = 0.001) (**Figure 3A**). Same results were obtained in parallel experiments using two other strains: Lt-RI325 and Lt-W (**Supplementary Figure 2**). These results confirmed the ability of *L. tarentolae* to dampen inflammasome activation. ASC-speck and ASC-diffuse images collected by AMNIS FlowSight representing WT THP-1dM cells stimulated with LPS + Nig and infected are shown in **Figures 3B, C**. In addition, the simultaneous detection of ASC expression and *Leishmania* cells after infection of THP-1 macrophages was carried out by immunofluorescence. As shown in **Supplementary Figure 3A**, when the cells were stimulated with LPS + Nig in the presence of the parasite, they mainly showed an ASC-diffuse pattern; on the contrary, when the cells were stimulated with only LPS + Nig, ASC specks were observed as red “spots” (**Supplementary Figure 3B**). Therefore, Lt-P10 markedly reduced the ASC-specks formation in response to stimulation with LPS + Nig.

### 3.4 Inflammasome-associated cytokines and caspase-1 in WT *L. tarentolae*-infected THP-1dMs and KO THP-1dM cells

**Figure 4:**
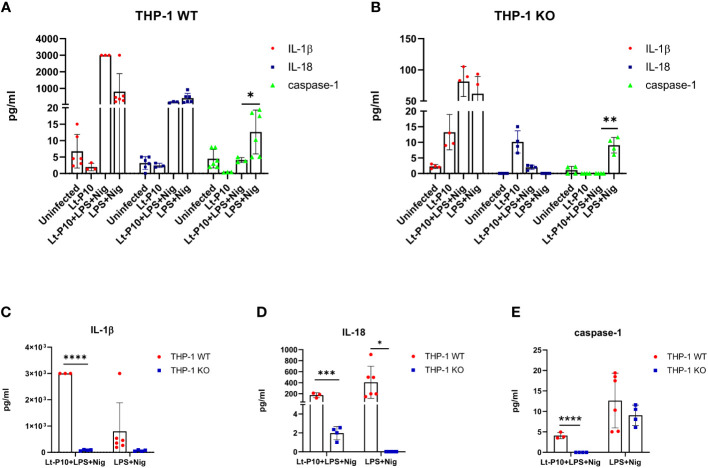
Cytokines and caspase-1 measured by immunoassay. Quantification of IL-1β, IL-18, and caspase-1 production in LPS + Nig–stimulated THP-1dM WT cells in the presence or absence of Lt-P10 **(A)** or LPS + Nig–stimulated THP-1dM ASC KO cells in the presence or absence of Lt-P10 **(B)** was performed by ELLA platform. Briefly, THP-1–derived macrophage cells were incubated with *L. tarentolae* promastigotes (10 parasites: one cell) for 4 h at 37°C to allow *Leishmania* internalization; cells were then incubated for 24 h, in medium supplemented with lipopolysaccharide (LPS) (10 ng/mL) plus Nigericin (Nig) (1.34 µM). Graphs related to each single cytokine comparing THP-1dM WT cells and THP-1dM KO cells are shown (**C–E**). Data are representative of three independent experiments and expressed as mean ± SD. Statistical significance differences are indicated. *p < 0.05, **p < 0.01, and ****p < 0.0001.

IL-1β, IL-18, and active caspase-1 were quantified by Automated Immunoassay System (ELLA) in the supernatants of LPS + Nig–stimulated and Lt-P10–infected or uninfected THP-1dMs WT cells. In LPS + Nig–stimulated WT THP-1dM cells, *L. tarentolae* infection resulted in a significant reduction (p = 0.01) of caspase-1 production, whereas that of IL-1β was increased (**Figure 4A**). IL-18 production was slightly reduced as well upon *L. tarentolae* infection, although differences did not reach statistical significance (**Figure 4A**).

In KO THP-1dM cells, production of active caspase-1 was significantly reduced by Lt-P10 infection (p = 0.004), whereas IL-1β production was increased, although not significantly (**Figure 4B**). Notably, IL-1β production was significantly reduced in Lt-P10 + LPS + Nig–stimulated THP-1dM KO compared to WT THP-1dM cells (p = 0.0001) (**Figure 4C**). Also, for IL-18 and caspase-1, there is a statistically significant reduction comparing WT THP-1 and KO THP-1 cells after infection with Lt-P10 (**Figures 4D, E**). Finally, human caspase-5 and caspase-8 production was evaluated by ELISA in supernatants of cells. The concentration of both proteins was below the limit of detection in WT THP-1dMs and in KO THP-1dMs in all experimental conditions (data not shown).

### 3.5 Cytokine production in *L. tarentolae*–infected WT THP-1dMs and KO THP-1dM cells

**Figure 5:**
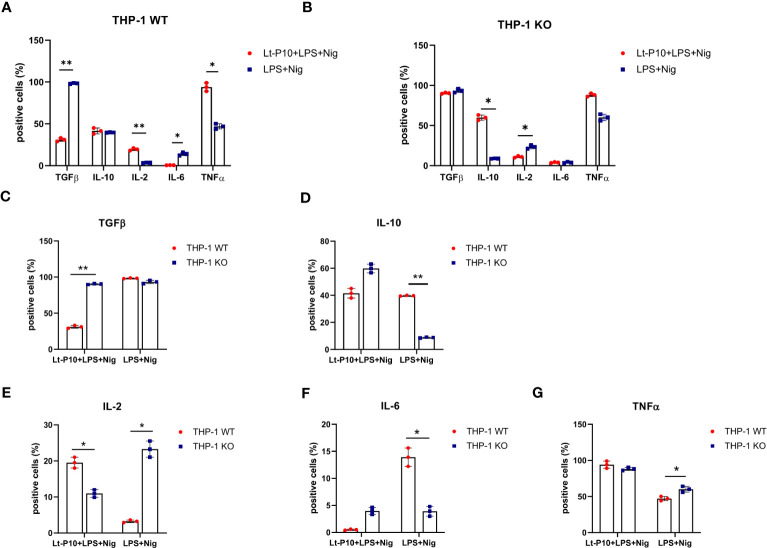
Immunity-associated factor quantification in WT and ASC-KO THP-1dMs. The percentage of TGF-β–, IL-10–, IL-2–, IL-6–, and TNF–expressing cells was determined by flow cytometry (Kaluza analysis software) in WT **(A)** and ASC-KO **(B)** cells. THP-1dMs were stimulated for 24 h with LPS + Nig in the presence or absence of Lt-P10. Then, cells were fixed, permeabilized, and stained with specific antibodies. Graphs related to each single cytokine comparing THP-1dM WT cells and THP-1dM KO cells are shown (**C–G**). Data are representative of three independent experiments and are expressed as mean ± SD. Statistical significance differences are indicated. *p < 0.05 and **p < 0.01.

TGF-β–, IL-10–, IL-2–, IL-6– and TNF-α–producing immune cells were examined by flow cytometry in WT THP-1dMs and in KO THP-1dM cells that were LPS + Nig–stimulated and Lt-P10–infected or uninfected. In WT THP-1dM cells, *L. tarentolae* infection resulted in a significant reduction of TGF-β (p = 0.01) and IL-6 (p = 0.03) production, whereas that of TNF (p = 0.03) and IL-2 (p = 0.01) was significantly increased. IL-10 production does not change upon infection (**Figures 5A, C–G**).

In KO THP-1dM cells, Lt-P10 infection induced a significant increase of IL-10 (p = 0.02) and a significant reduction of IL-2 (p = 0.03); TGF-β production was only marginally modified (**Figures 5B, C–G**). Representative histograms were shown in supplementary materials (**Supplementary Figures 4**, **5**).

## 4 Discussion

Several studies have investigated the mechanisms involved in the development of protective immunity in *Leishmania* spp. infections. Indeed, the identification and description of immune correlates of protection in leishmaniases is important, as it would possibly lead to the design of novel strategies to formulate vaccines and immune-therapeutics against *Leishmania* infections (29). In this context, the possible connection between *Leishmania* infection and the activation or inhibition of NLRP3 inflammasome formation has been investigated for pathogenic species (19–22).

Notably, the capability of *Leishmania* spp. to downregulate the NLRP3 inflammasome activation could be extremely important from a therapeutic point of view, as excessive inflammasome activation has been shown to play a key role in the pathogenesis of several diseases in humans (30–42). Humans, of course, cannot be infected with pathogenic *Leishmania* species, in order to contain inflammation. However, the administration of *Leishmania* strains that are not pathogenic to humans (or derived molecules or fractions) could be hypothesized, in inflammatory conditions. To this end, we determined whether strains belonging to a non-pathogenic *Leishmania* species could downregulate NLRP3 activation and the subsequent inflammatory response in human cells. The use of *L. tarentolae* to dampen the NLR signaling pathway might lead to practical applications, as this species is already investigated as a vaccine platform and as source of molecules suitable to be administered to humans and animals (3, 4). In the current study, the effect of *L. tarentolae* on the NLRP3 inflammasome activation has been investigated using the human monocyte cell line THP-1 both WT and KO for ASC. In particular, both WT and KO THP-1 cells, differentiated into macrophages, were exposed to three strains of *L. tarentolae* (two field-isolated strains and one laboratory strain), after stimulation with LPS + Nig.

*L. tarentolae* has already been shown to be suitable to be phagocyted by human dendritic cells (13); our results indicate that this parasite can also be engulfed by THP-1–derived macrophage cells, in coherence with previous results with *L. infantum* on the same cell line (22), or in monocyte-derived, canine macrophages (43); in addition, *L. tarentolae* has also been shown to be phagocyted by canine macrophages (44). Notably, in WT THP-1–derived macrophages, the percentage of ASC-speck–positive cells was significantly reduced upon *L. tarentolae* infection. This resulted in a reduced production of inflammatory protein caspase-1 downstream of inflammasome activation. As for IL-18 production by WT THP-1 cells infected with Lt-P10, cytokine in supernatant showed a reduction that was not significant. Reduction of IL-18 expression was, however, significant at the level of the mRNA. The classic assembly of NLRP3 inflammasome takes place after the recognition of PAMPs and danger-associated molecular patterns and occurs in two steps: 1) a *priming* signal that can be induced by Toll-like receptors and leads to NF-κB activation, resulting in the upregulation of the inflammasome components pro–IL-1β, sensors, and caspase-1 (45–47); and 2) a *second* signal that allows the NLRs oligomerization following the assembly of NLR, ASC, and caspase-1, with the consequent cleavage of pro–IL-1β and pro–IL-18. Human monocytes can also use an alternative NLRP3 inflammasome activation pathway that proceeds in the absence of signal 2 activation and enables IL-1β secretion in the absence of caspase-1 triggering. In this case, NLRP3 signaling relies on caspase-8, and the final result is inflammasome activation without pyroptosome formation (48). Notably, in this case, caspase-8 activation can also induce IL-1β maturation independently of the action of caspase-1 (49). Another non-canonical activation of NLRP3 is driven by the LPG of *Leishmania*, which activates mice caspase-11 and human caspase 4/5 (20). Results herein show that caspase-5 and -8 production were not detectable in supernatants of *Leishmania* infected and LPS + Nig–stimulated cells, suggesting that alternative inflammasome activation modalities are not active in this *in vitro* model.

The inflammasome is activated in *Leishmania* infection (50–52); however, the role of inflammasomes and IL-1β in the immune response to *Leishmania* parasites is very controversial, mainly because of differences in cultural conditions. Thus, the use of different *Leishmania* species, developmental stages, and infection models lead to results that are difficult to compare. Overall, previous data suggest that different parasite strains vary in modulating inflammasome activation. Hence, *L. donovani* and *L. tropica* were reported not to induce IL-1β production and to downregulate Interferon (IFN)-primed human or LPS-primed murine peritoneal macrophages (53–55). Other results suggested that infection of human (56–58) or murine phagocytes by *L. major* cause a parasite-mediated dysregulation of IL-1β on a transcriptional level. Conversely, other studies (58, 59) indicate that *L. major* infection increases inflammasome activity and cytokine production in long-term infections. Recent data also suggest that inflammasomes and IL-1β are involved in the control of *L. amazoniensis* infection of C57Bl/6 mice, as shown by *in vitro* and *in vivo* studies using mice deficient in IL-1β production (including caspase-1 and NLRP3 KO mice) (51).

It should be noted that several studies have indicated IL-1β production as associated with the early stages of infection by *Leishmania* spp. (60–62). As for IL-18, in *L. major*–infected BALB/c mice, this cytokine was shown to upregulate IL-4 production, favoring the persistence of infection. These results are supported by the observation that IL-18 neutralization in this animal model resulted in greater resistance to infection by decreasing IL-1β and IL-18 production and by controlling parasitic growth (59).

IL-1β is a key cytokine in inflammation, produced by mononuclear phagocytes. It is produced as an inactive cytoplasmic precursor and is then cleaved by caspase-1 to become biologically active, in a process that can be either dependent or independent of inflammasome activation (63). As reported by Guey et al. (64) the NLRP3 inflammasome requires the presence of ASC to activate caspase-1. Indeed, there is solid evidence that, in response to NLRP3 activators, IL-1β secretion is abolished and pyroptosis is strongly impaired in ASC-KO macrophages. Herein, after *L. tarentolae* infection, we observed elevated levels of IL-1β in WT cells, whereas the production of this cytokine did not change significantly in ASC-KO. Notably, after infection, IL-1β expression in ASC-KO was significantly lower than in WT cells. Unexpectedly, a lasting production of caspase-1 was observed in ASC-KO cells. Coherently with this result, other inflammasomes, differently from NLRP3, can efficiently activate caspase-1 in the absence of ASC and caspase-1 autoproteolysis. This has actually been observed in mice (64), but this mechanism is not completely clear in humans (65, 66). However, unlike the other NLRPs, human NLRP1 and CARD8 inflammasomes have a C-terminal extension containing a function-to-find domain (FIIND) and a CARD. In particular, FIIND autoproteolysis is required for NLRP1 inflammasome activation (67); the C-terminal CARD, and not the N-terminal PYD, that is required in mice, recruits ASC to form inflammasome (66, 68). The human NLRP1 (hNLRP1) is encoded by a single gene, differently from its murine counterpart, which is encoded by three paralogues (NLRP1a, NLRP1b, and NLRP1c); hNLRP1 is the only NLR that can potentially be degraded through a process of post-translational autoproteolysis, at position Ser1213 of the FIIND (67), that results in the C-terminal and N-terminal portions remaining non-covalently linked. Although only a fraction of the total NLRP1 protein undergoes autoproteolysis (69), this event is essential for subsequent NLRP1 activation, self-oligomerization, and inflammasome assembly.

Interestingly, some CARD domains can directly recruit pro-CASP1 independent of ASC (65, 68, 70). CARD8, instead, has a similar-human NLRP1-FIIND-CARD regions, and it undergoes FIIND autoproteolysis and the C-terminal CARD domain can form an inflammasome. However, unlike human NLRP1, the CARD8 can directly interact with the CARD of pro-CASP1 and does not form an ASC-containing platform (65, 71). Furthermore, CARD8 is expressed in humans but not in mice, a model in which the issue has not yet been deeply investigated.

Regarding the different effects of *L. tarentolae* stimulation in WT and KO cells, the absence of ASC protein in KO cells is probably responsible for the reduction of IL-1β, IL-18, and caspase-1 production. Considering CARD8 and NLRP1, we could hypothesize that epigenetic regulation may be involved in the observed pattern. In general, in the absence of ASC, epigenetic regulation might play a role in modulating the expression of genes that contribute to inflammasome formation. Indeed, current evidence supports the idea that DNA methylation, histone modifications, or miRNAs play a role in regulating the expression of inflammasome components (72, 73). For example, inflammasome-related genes have been shown to be demethylated during macrophage differentiation and monocyte activation (74). Furthermore, miRNAs have been shown to participate in the post-transcriptional regulation of NLRP1 (75).

In terms of cytokine expression, our study was mainly focused on IL-1β and IL-18, considering the role of the NLRP3 inflammasome in the activation of their precursors. In addition, we determined the expression of five further cytokines that are produced by macrophages, characterized by anti-inflammatory (IL-10 and TGF-β) or pro-inflammatory (TNF-α, IL-2, and IL-6) properties. These cytokines have indeed been shown to be associated with different outcomes of the infection in different forms of leishmaniasis (21). In our experiments, an increase of IL-2–producing immune cells was recorded by flow cytometry analyses after Lt-P10 + LPS + Nig infection of WT THP 1dM cells; it is known that IL-2 is involved in controlling inflammation by inhibiting Th17 cells and IL-6 receptor (76). However, an increase of TNF-α and a decrease of TGF-β-producing cells have been also revealed in our experiments. On the contrary, in THP-1 ASC KO cells, after Lt-P10 co-incubation, we observed a decrease in IL-2–producing cells and an increase of those producing IL-10. Overall, these results support the hypothesis that *L. tarentolae* could play a role in the control of inflammation, even in the absence of ASC activation.

Results herein show that infection of human cells with the non-pathogenic *L. tarentolae* interferes with NLRP3 formation. Although a limitation of this *in vitro* model is that it fails to replicate the complexity of the cellular and intercellular environment associated with the mammalian immune system, our results suggest that a non-pathogenic *Leishmania* species could be investigated as an anti-inflammatory agent or as a source of anti-inflammatory molecules.

## Data availability statement

The original contributions presented in the study are included in the article/**Supplementary Material**, further inquiries can be directed to the corresponding author/s.

## Ethics statement

Ethical approval was not required for the studies on humans in accordance with the local legislation and institutional requirements because only commercially available established cell lines were used.

## Author contributions

FLR: Investigation, Formal Analysis, Writing – original draft, Conceptualization, Methodology. IV-B: Conceptualization, Investigation, Writing – original draft, Writing – review & editing. MS: Writing – review & editing, Supervision. IM: Methodology. GC: Writing – original draft, Investigation. AH: Writing – original draft, Investigation. FP: Writing – original draft, Formal Analysis. DO: Writing – review & editing. SE: Writing – review & editing, Funding acquisition. CB: Writing – original draft, Writing – review & editing, Funding acquisition. MC: Writing – review & editing, Funding acquisition, Supervision.

## Supplementary material

Supplementary Figure 1 Phagocytosis of *L. tarentolae* strains by THP-1 cells after 4h of incubation. THP-1 WT macrophages were incubated with Lt-P10, Lt-RI325 and Lt-W at 1:10 ratio (THP-1: *Leishmania*). Cells were then fixed, stained with Giemsa solution and observed under an optical microscope (100X). Giemsa smears of Lt-P10 **(A)**, Lt-RI325 **(B)** and Lt-W (**C**) incubated with THP-1 WT are shown. Red arrows indicate internalized promastigotes inside the cells. Bar: 10 μm. The percentage of infected macrophages (infection rate) was determined for each Lt strain incubated with THP-1 WT and for Lt-P10 incubated with THP-1 KO. Results are expressed as mean ± deviation standard (SD). No significant differences were observed (p ≥ 0.05) **(D)**. Quantification of lactate dehydrogenase (LDH) released by THP-1 WT cells after infection with Lt-P10, Lt-RI325 and Lt-W is expressed as percentage of internal LDH positive control (PC) **(E)**. Determination was carried out on uninfected cells and cells infected with the three *L. tarentolae* strains. Bars show mean ± SD. No significant differences were observed (p > 0.05).

Supplementary Figure 2 ASC-speck quantification in THP-1dM WT cells after infection with Lt-RI325 or Lt-W. Formation of ASC-speck in THP-1dM cells stimulated with LPS + Nig and incubated with or without one of the two field-isolated strains (Lt-RI325 or Lt-W) or unstimulated (MED) are shown in the graph. Data are representative of three independent experiments and expressed as mean ± SD. Statistical significance differences are indicated. * p < 0.05; ** p < 0.01; *** p < 0.001.

Supplementary Figure 3 Simultaneous detection of ASC expression and *Leishmania* cells through immunofluorescence assay. THP-1dM cells incubated with Lt-P10 and stimulated with LPS + Nig were collected, cytocentrifuged for 5 min at 500 rpm on a slide, fixed with PFA in PBS and stained with PE-anti human ASC mAb (RED), mouse anti-*Leishmania* Ab, Alexa Fluor 488-conjugated anti-mouse (GREEN). Cells were then mounted with ProLong Gold Antifade Mountant with DAPI (BLUE). ASC-speck was not detected in Lt-P10–infected cells **(A)**; conversely in infected cells **(B)**. Red arrows indicate *Leishmania* cells (green), while yellow arrows indicate ASC speck. Fluorescent results were observed under a fluorescence microscopy Leica DMi8 with Thunder imaging systems with 40X objective.

Supplementary Figure 4 Determination of cytokine-expressing THP-1 WT cells by flow cytometry. Representative images of the percentage of TGF-β, TNF, IL-6, IL-2, and IL-10 expressing cells were determined by flow cytometry in WT THP-1dMs stimulated with LPS + Nig in the presence **(A)** or in absence **(B)** of Lt-P10. Cells after the incubation with Lt-P10 were stimulated with LPS and Nigericin. After 24h cells were fixed, permeabilized and stained with specific antibodies. Data are representative of three independent experiments and are expressed as histograms for each protein.

Supplementary Figure 5 Determination of cytokine-expressing THP-1 KO cells by flow cytometry. Representative images of the percentage of TGF-β–, TNF-α–, IL-2–, IL-10–, and IL-6–expressing cells were determined by flow cytometry in KO THP-1dMs stimulated with LPS + Nig in the presence **(A)** or in absence **(B)** of Lt-P10. Cells after the incubation with Lt-P10 and LPS-Nig treatment were fixed, permeabilized and stained with specific antibodies. Data are representative of three independent experiments and are expressed as histograms for each protein.

Supplementary Table 1 260/230 Absorbance (A) ratio. mRNA quantification in THP-1dM WT cells unstimulated and after incubation with *L. tarentolae* laboratory strain P10 (Lt-P10) or with lipopolysaccharide (LPS)+Nigericin (Nig) or with Lt-P10 + LPS + Nig.
