# Supplementary material for: The non-pathogenic protozoon Leishmania tarentolae interferes with the activation of NLRP3 inflammasome in human cells: new perspectives in the control of inflammation
Source: Front Immunol. 2024 Apr 19;15:1298275. doi: 10.3389/fimmu.2024.1298275 (PMC11066211; doi:10.3389/fimmu.2024.1298275)
Supplement: Supplementary Figure 1 — Phagocytosis of L. tarentolae strains by THP-1 cells after 4h of incubation. THP-1 WT macrophages were incubated with Lt-P10, Lt-RI325 and Lt-W at 1:10 ratio (THP-1: Leishmania). Cells were then fixed, stained with Giemsa solution and observed under an optical microscope (100X). Giemsa smears of Lt-P10 (A), Lt-RI325 (B) and Lt-W (C) incubated with THP-1 WT are shown. Red arrows indicate internalized promastigotes inside the cells. Bar: 10 μm. The percentage of infected macrophages (infection rate) was determined for each Lt strain incubated with THP-1 WT and for Lt-P10 incubated with THP-1 KO. Results are expressed as mean ± deviation standard (SD). No significant differences were observed (p ≥ 0.05) (D). Quantification of lactate dehydrogenase (LDH) released by THP-1 WT cells after infection with Lt-P10, Lt-RI325 and Lt-W is expressed as percentage of internal LDH positive control (PC) (E). Determination was carried out on uninfected cells and cells infected with the three L. tarentolae strains. Bars show mean ± SD. No significant differences were observed (p > 0.05). [file DataSheet_1.pdf]

Figure S1

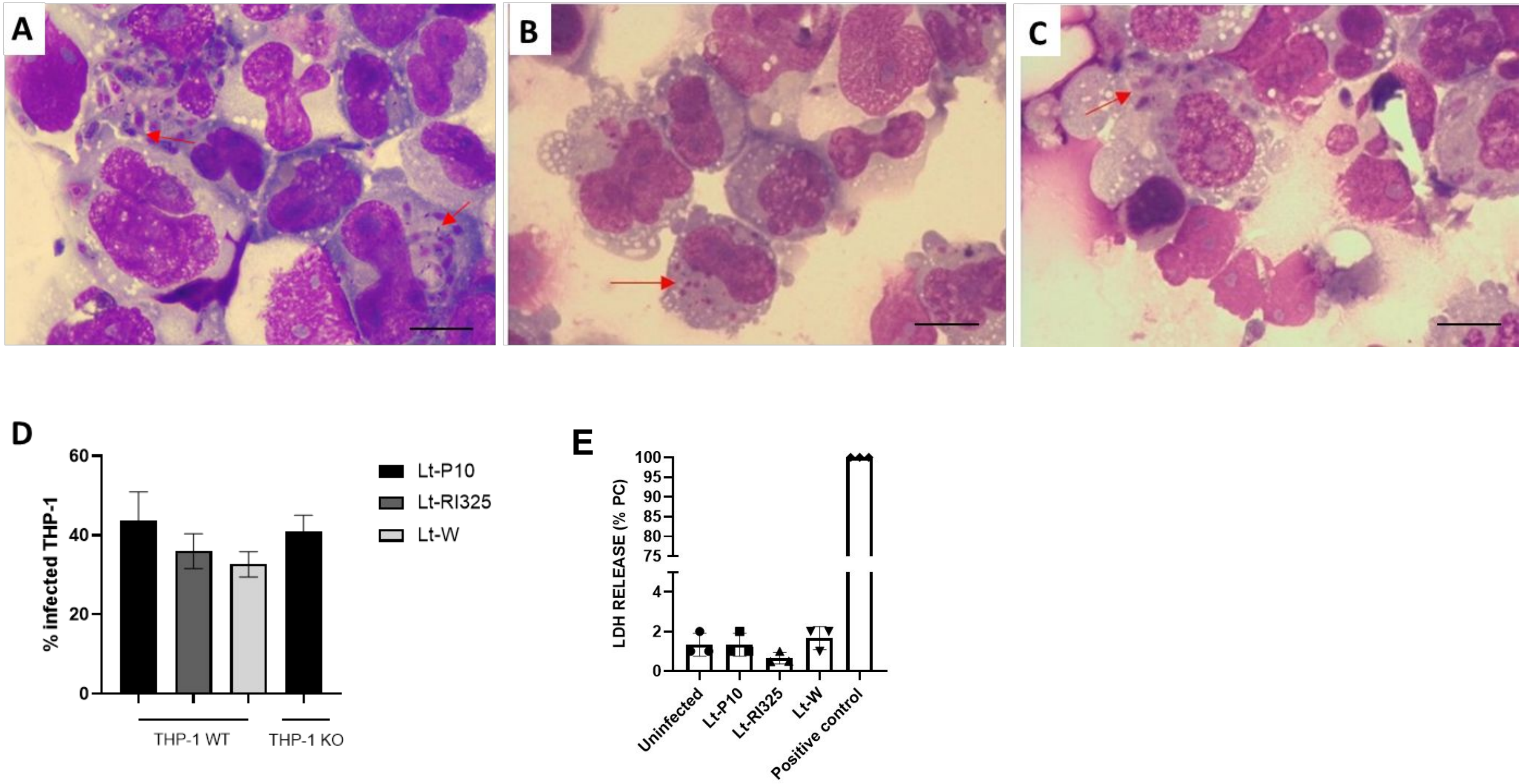
A
B
C
D
60
40
20
0
% infected THP-1
THP-1 WT
THP-1 KO
Lt-P10
Lt-RI325
Lt-W
E
100
95
90
85
80
75
4
2
0
LDH RELEASE (% PC)
Uninfected
Lt-P10
Lt-RI325
Lt-W
Positive control

Figure S2

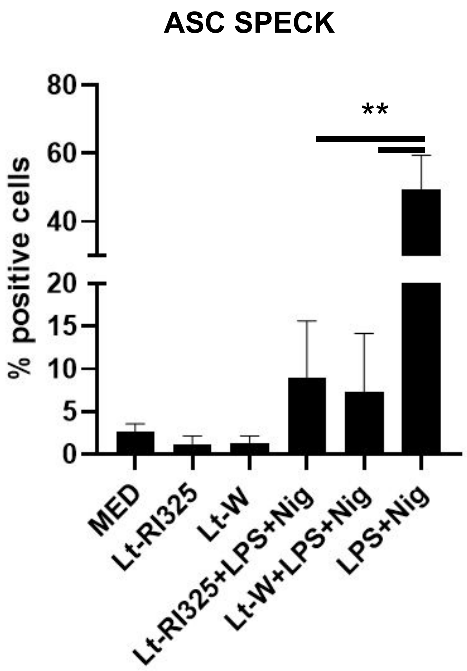
ASC SPECK
% positive cells
80
60
40
20
15
10
5
0
**
MED
Lt-RI325
Lt-W
Lt-RI325+LPS+Nig
Lt-W+LPS+Nig
LPS+Nig

Figure S3

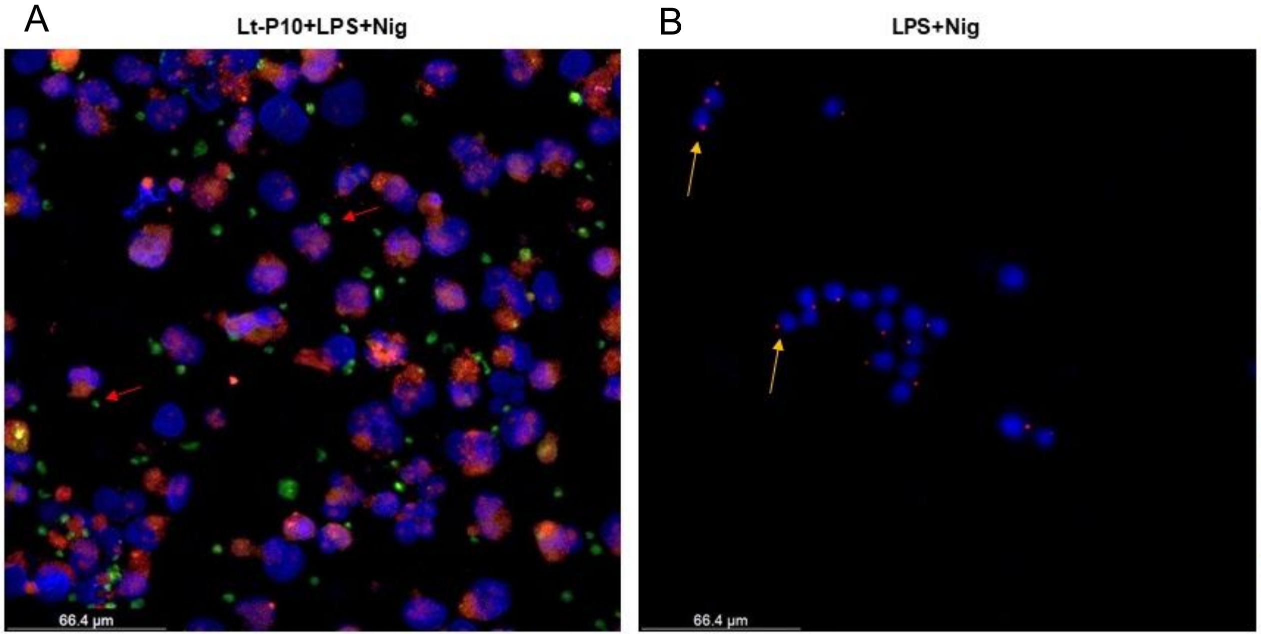

Figure S4

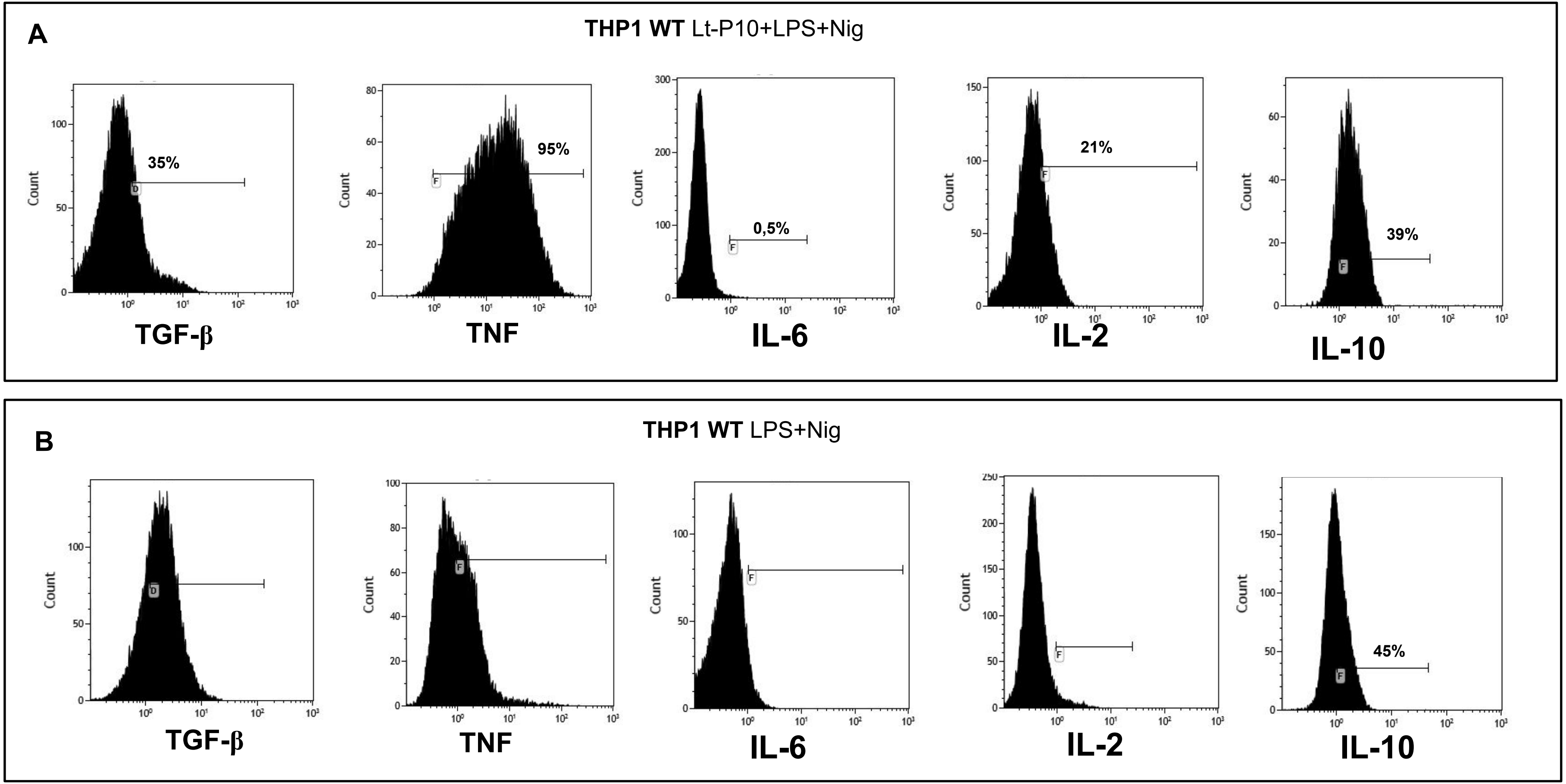
A
THP1 WT Lt-P10+LPS+Nig
Count
35%
TGF-β
95%
TNF
0,5%
IL-6
21%
IL-2
39%
IL-10
B
THP1 WT LPS+Nig
Count
TGF-β
TNF
IL-6
IL-2
45%
IL-10

Figure S5

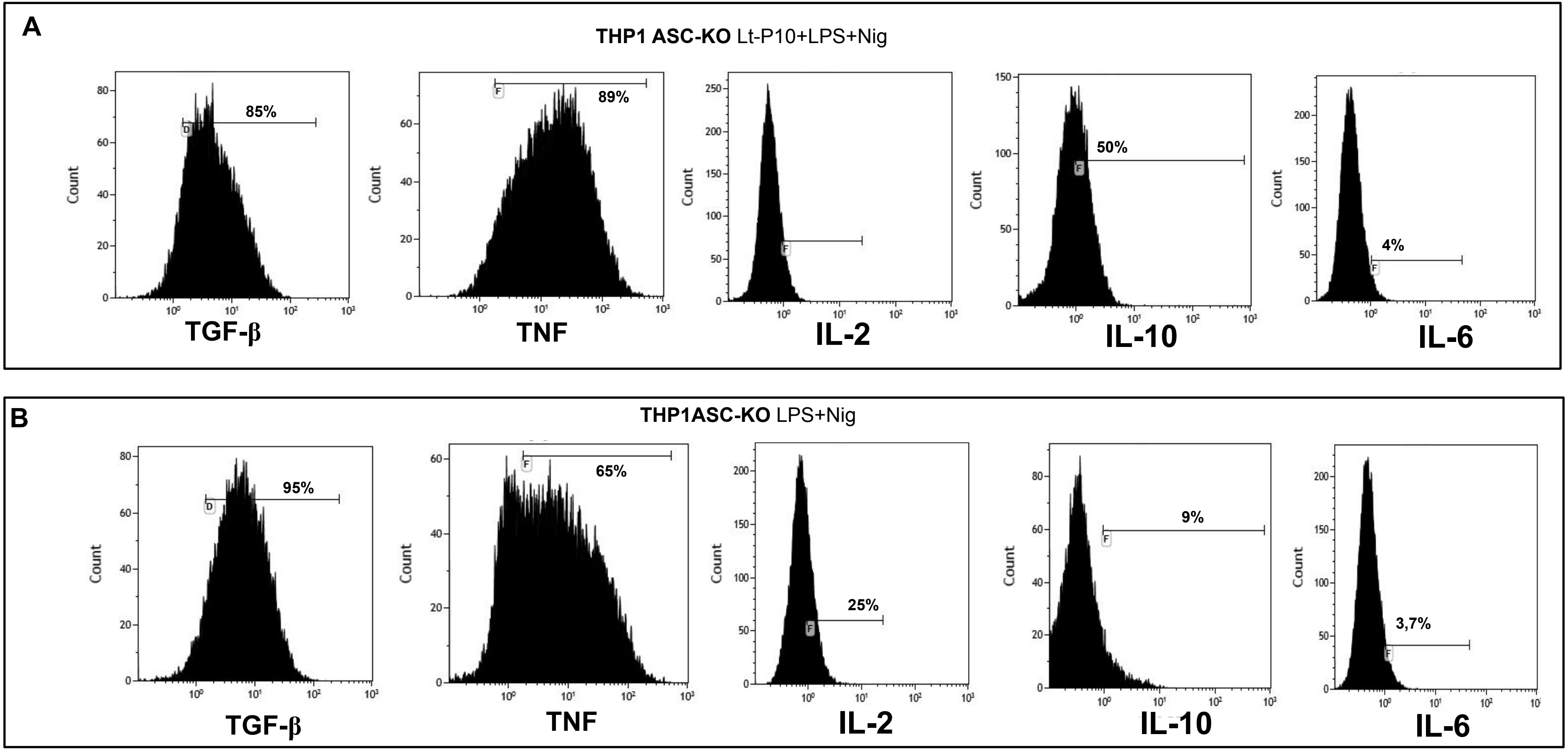

A
THP1 ASC-KO Lt-P10+LPS+Nig
85%
89%
50%
4%
TGF-β
TNF
IL-2
IL-10
IL-6
Count
B
THP1ASC-KO LPS+Nig
95%
65%
25%
9%
3,7%
TGF-β
TNF
IL-2
IL-10
IL-6
Count

Table S1

		A260/A230 (nm)		
Sample	condition	exp I	exp II	exp III
THP-1 dM	unstimulated	1,911	1,962	2,01
THP-1 dM	Lt-p10	1,955	1,896	1,921
THP-1 dM	LPS+Nig	1,798	1,851	1,801
THP-1 dM	Lt-P10+LPS+Nig	1,796	1,856	1,852
